# Characteristics and outcome of patients with left atrial appendage closure in China: a single-center experience

**DOI:** 10.1186/s12872-023-03651-8

**Published:** 2024-02-14

**Authors:** Jingrui Zhang, Changyi Li, Lu Zhou, Caihua Sang, Songnan Li, Changsheng Ma

**Affiliations:** 1 Department of Cardiology, Beijing Anzhen Hospital affiliated Capital Medical University, No. 2 Anzhen Road, Chaoyang District, Beijing, 100026 China; 2 National Clinical Research Center for Cardiovascular Diseases, Beijing, China

**Keywords:** Atrial fibrillation, Left atrial appendage closure, Long-term follow up

## Abstract

**Background:**

Clinical characteristics and long-term data on the safety and efficacy of LAAC in preventing cerebrovascular accident and thromboembolism among Chinese patients with non-valvular AF (NVAF) remain limited.

**Methods:**

Data of consecutive NVAF patients who underwent LAAC at Beijing Anzhen Hospital, Capital Medical University, from June 1, 2014, to December 31, 2021, were collected and analyzed retrospectively. The primary effectiveness endpoint was the composite endpoint of stroke/transient ischemic attack, systemic embolism, and death from cardiovascular causes. The primary safety endpoint is the severe bleeding defined by the LAAC Munich consensus.

**Results:**

Of the 222 patients enrolled, the mean age was 66.90 ± 9.62 years, with a majority being male (77.03%). Many patients are non-paroxysmal AF (71.19%) with a median duration of AF of 4.00 years. The mean CHA2DS2-VASc score was 3.78 ± 1.49, and the mean HAS-BLED score was 1.68 ± 0.86. Thromboembolic events (76.58%) were the most common indication for LAAC. The device, technical, and procedural success rates were 98.65%, 98.65%, and 93.69%, respectively. The anticoagulation continuation rate was 56.36%, 31.25%, and 22.60% at 3-, 6- and 12 months post-procedure, respectively. Throughout a mean 2.81 years of follow-up, the incidence of the primary efficacy endpoint was 4.27 per 100 patient-years, predominantly attributable to stroke/TIA (3.12 per 100 PYs). Five patients experienced major bleeding during the follow-up period. Post-procedure imaging revealed minimal complications, with only one substantial peri-device leak. Device-related thrombus occurred in 2.33% of patients, resolving with anticoagulation.

**Conclusion:**

The study demonstrates that LAAC is a safe and effective alternative option for Chinese patients with AF, with a high success rate, few complications as well as fewer long-term adverse outcome events.

**Supplementary Information:**

The online version contains supplementary material available at 10.1186/s12872-023-03651-8.

## Introduction

The increasing incidence of atrial fibrillation (AF) constitutes a heavy burden on society globally, with an estimated 8 million people suffering from AF in China, taking up a large part of scarce medical resources [1, 2]. Patients with nonvalvular AF have a 3- to 5-fold increased risk of stroke and systemic thromboembolic events in the absence of anticoagulation therapy [3]. Compared with non-AF-related strokes, disability and mortality rates are higher in AF-related strokes, and the medical costs are 1.5 times higher than those of non-AF-related strokes [4]. Anticoagulation is an effective way to reduce the likelihood of AF-related stroke [5]. However, in the real-world setting, despite the wide availability of direct oral anticoagulant (DOAC), adherence to OAC therapy remains poor among AF patients [6]. The discontinuation of OAC in several randomized studies is high [7–9]. Moreover, in patients with AF at increased risk for bleeding, the prescribing rate of anticoagulants was low due to concerns about the potential for bleeding.

TEE and other imaging modalities supported that more than 90% of left atrial thrombi were in the left atrial appendage (LAA) among patients with non-valvular AF (NVAF) [10, 11]. Based on this theory, the use of mechanical means to seal the LAA is expected to prevent stroke in AF patients without increasing the risk of bleeding. The safety and efficacy of LAA closure (LAAC) in reducing the risk of stroke and systemic thromboembolic events have been supported in several multicenter randomized clinical trials and registry trials [12, 13]. The RECORD study conducted by Tao et al. included data from 39 hospitals in China with 3096 patients enrolled. The results showed that the procedural success rate of LAAC was higher than 97%, with a low rate of perioperative complications and short-term adverse events (0.52% for the composite endpoint consisting of death, stroke, and systemic thromboembolism, and 1.23% for life-threatening bleeding) [14]. However, this study just reported results on the in-hospital and 30-day follow-up data. Data on long-term follow-up after LAAC procedures for atrial fibrillation in China are still limited. The purpose of this study was to provide the experience of LAAC surgery in our center and the long-term outcomes of patients.

## Methods

### Patient population and study design

This is a retrospective analysis of consecutive NVAF patients aged 18 years and older who underwent the LAAC procedure at Beijing Anzhen Hospital affiliated Capital Medical University between June 1, 2014, and December 31, 2021. All patients enrolled either had at least one contraindication to OAC or were unwilling to accept long-term OAC therapy and signed informed consent before the LAAC procedure. Data about baseline characteristics, procedure, imaging, anti-thrombotic regimen, and outcomes in hospital and post-charge were collected from medical records and registry database. This study was approved by our institution’s ethical committee and was conducted in accordance with the Declaration of Helsinki. Informed Consent was obtained from all participants involved in the study.

### Peri-procedural imaging and device implantation strategy

Oral anticoagulants were started 4 weeks before the procedure, except for whom blood thinning therapy were contraindicated. One to two days before the procedure, all patients underwent transesophageal echocardiography (TEE) or cardiac CT angiography (CCTA) imaging to determine the anatomy of LAA and surrounding structures and rule out thrombus in the LA and LAA. In the previous years of starting LAAC device implantation in our center, the procedures were conducted under the guidance of both TEE and fluoroscopy. All cases were subject to general anesthesia by intravenous propofol infusion (2-2.5 mg/kg). Later, we adopted the so-called minimalist approach in January 2020 as experience enriched and accumulated to reduce respiratory depression and post-operative delirium. The detailed steps are described elsewhere [15, 16]. The implantation steps are similar to the EHRA/EAPCI expert consensus statement, except for the omission of the TEE [17]. In addition, the detailed procedural steps are also described in the supplementary material. Furthermore, the comprehensive procedure details are also outlined in the supplementary material.

A peri-procedural anticoagulation regimen adopted the low molecular weight heparin bridging strategy in the previous years. Also, since January 2020, the anticoagulation regimen was switched to the uninterrupted dabigatran strategy. For patients with drug-refractory AF, the decision to perform catheter ablation of AF is made by the cardiologist and the patient after a shared discussion.

### Post-procedural anti-thrombotic regimen

After the procedure, all patients were treated with anticoagulants if they had no contraindications for the first 45 days. If the patient underwent catheter ablation of AF at the same time, anticoagulation duration lasted for 3 months. DOACs would be the first choice for these patients. For patients with a high risk of a thromboembolic event (CHA2DS2-VASc score of 2 or higher [3 or higher in females] or stroke on OAC), low-dose aspirin (100 mg/d) and/or clopidogrel (75 mg/d) was added to the anti-thrombotic regimen in addition to the anticoagulant. Dual antiplatelet therapy (DAPT) with aspirin (100 mg/d) plus clopidogrel (75 mg/d) were only given to patients at high risk of bleeding (HASBLED score of 3 or higher, or those with absolute contraindications to long-term OAC therapy) initially. Device stability and position, device-related thrombus (DRT), and peri-device leakage (PDL) were reevaluated using TEE or CCTA 45–90 days post-procedure and repeated in the event of an unexpected event during follow-up. If the outcome was satisfactory (PDL < 5 mm and no DRT), anticoagulation was discontinued and replaced by DAPT for six months, followed by lifetime treatment with low-dose aspirin if the results of re-evaluation were satisfactory as well.

### Primary and secondary endpoints

Patients were followed up by telephone and in-office interviews. Follow-up visits were at 45 days, 3, 6, and 12 months, and every 6 months thereafter. The primary effectiveness endpoint is the composite endpoint of transient ischemic attack (TIA), ischemic stroke, systemic embolism, and death from cardiovascular causes. The primary safety endpoint is the composite endpoint of hemorrhagic stroke and severe bleeding. Secondary endpoints were each component of the primary effectiveness and safety endpoint. Endpoint events were defined according to the LAAC Munich consensus document. The technical success (defined as successful delivery and deployment of the LAAC device into the LAA), occlusion success (defined as PDL < 5 mm assessed by TEE or CCTA at 45 days after LAAC), and procedural success (defined as technical success without any major adverse events) were also reported in our study.

### Statistical analysis

Quantitative data were expressed as mean ± standard deviation (SD), and categorical data were expressed as numbers and percentages. Continuous variables conforming to normal distribution were expressed as mean ± standard deviation (SD), and continuous variables with non-normal distribution were expressed as median (interquartile range, IQR). Categorical variables were expressed as frequencies (percentages). Student’s t test or Fisher’s exact tests were employed for comparisons between groups. The incidence of stroke and major bleeding events was calculated using Kaplan-Meier survival analysis. Statistical analyses were performed using the R software version 4.2.1.

## Results

### Baseline characteristics

**Fig. 1:**
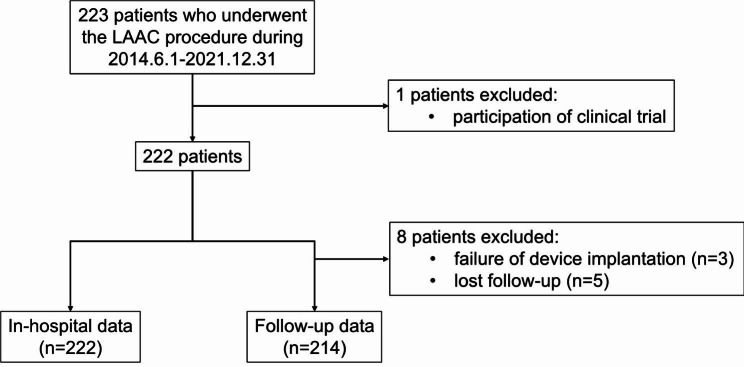
The study flowchart

**Fig. 2:**
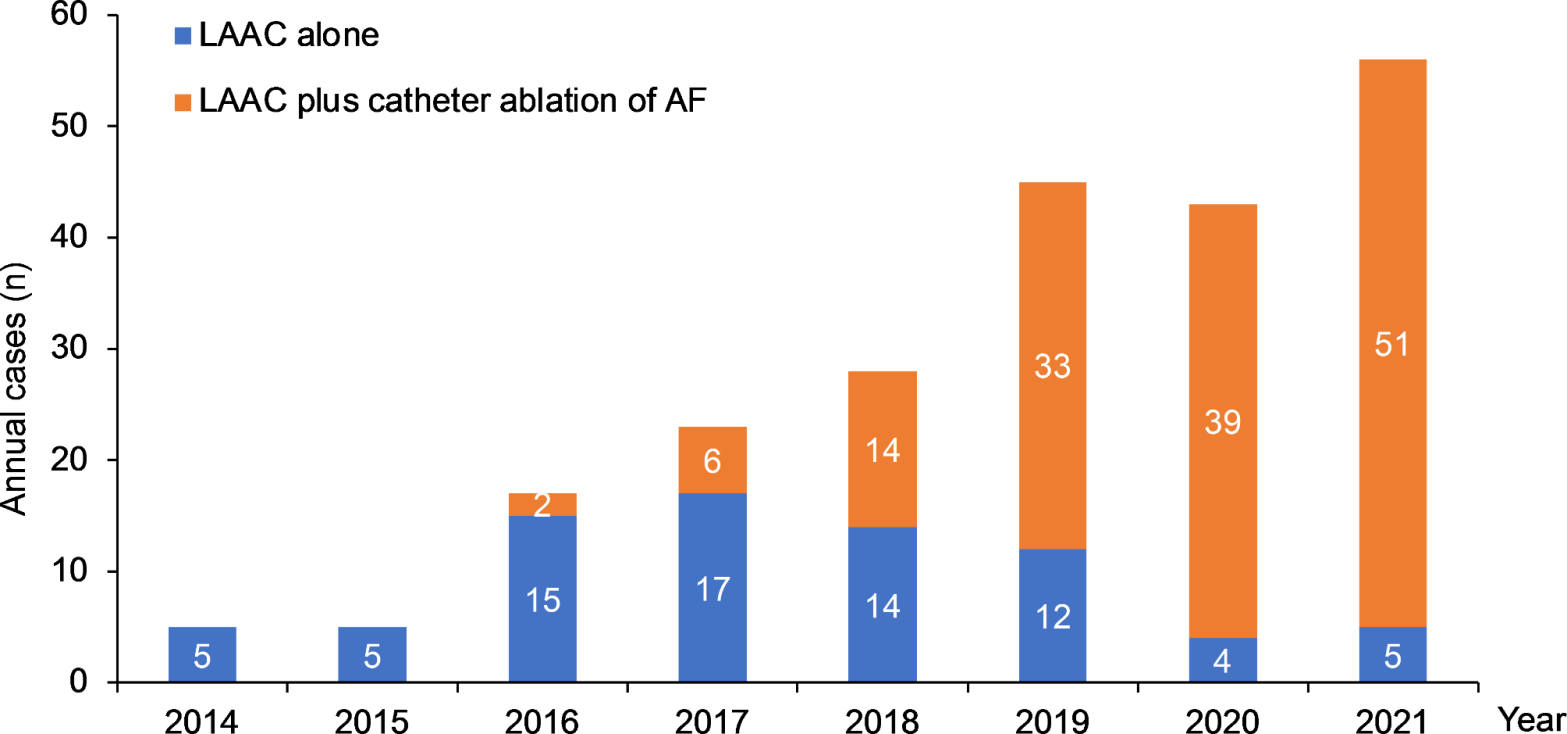
The annual cases of LAAC. LAAC, left atrial appendage closure; AF, atrial fibrillation

A total of 223 AF patients who underwent the LAAC procedure were retrieved from the medical records. One patient was excluded from the implantation of the occluder being tested in a clinical trial. We report surgical and in-hospital data for the remaining 222 patients. A total of 214 individuals were followed-up till the inception of the study. The study flowchart is shown in Fig. 1. In 3 cases, the LAAC devices were not successfully implanted. Two failed procedures were attributed to the small diameter of the LAA ostium and insufficient working depth. In the other case, the unsuccessful implantation was due to the oversized LAA ostium diameter. The mean age was 66.90 ± 9.62 years old, and more than half were elderly (60.92%). Males accounted for 77.03%. The mean BMI was 26.10 ± 3.90 kg/m^2^. Many patients are non-paroxysmal AF (persistent or long-standing, 71.19%) with a median duration of AF of 4.00 years. The most frequent comorbidities among patients were hypertension (70.27%), followed by diabetes (27.48%), coronary artery disease (26.13%), peripheral artery diseases (16.22%), and heart failure (11.71%). The mean CHA2DS2-VASc score was 3.78 ± 1.49, and the mean HAS-BLED score was 1.68 ± 0.86. One hundred seventy patients (76.58%) had a previous history of thromboembolic events (stroke/transient ischemic attack and systemic embolism), and 53 patients (24.32%) had a prior history of the Bleeding Academic Research Consortium (BARC) type 3a-5 bleeding event. 166 patients (74.77%) were taken OAC before undergoing LAAC procedure. In addition to the larger left and right atrium, the remaining heart chambers and mean left heart ejection fraction were normal. The detailed baseline demographic and clinical characteristics are summarized in Table 1. The number of cases per year for LAAC and LAAC combined with AF catheter ablation is shown in Fig. 2.

**Table 1:** Baseline demographic and clinical characteristics

Characteristics	All patients (n = 222)
Age, years (mean ± sd)	66.90 ± 9.62
< 65, n (%)	85 (39.10%)
65–75, n (%)	94 (41.82%)
> 75, n (%)	43 (19.10%)
Male, n (%)	171 (77.03%)
BMI (kg/m2)	26.10 ± 3.90
AF Duration, year	4 (1—10)
nPAF, n (%)	158 (71.17%)
Pre-procedure OAC use, n(%)	166 (74.77%)
Comorbidity	
Hypertension, n (%)	156 (70.27%)
Heart Failure, n (%)	26 (11.71%)
HCM, n (%)	11 (4.95%)
Diabetes mellitus, n (%)	61 (27.48%)
Stroke/TIA, n (%)	155 (69.82%)
Systemic thromboembolism, n (%)	15 (6.76%)
CAD, n (%)	58 (26.13%)
PAD, n (%)	36 (16.22%)
PCI, n (%)	23 (10.36%)
CABG, n (%)	7 (3.15%)
CKD, n (%)	13 (5.86%)
Dialysis, n (%)	3 (1.35%)
Previous LAA thrombus, n (%)	18 (8.49%)
CHA2DS2-VASc score	3.78 ± 1.49
0, n (%)	3 (1.35%)
1, n (%)	11 (4.95%)
2, n (%)	31 (13.96%)
3, n (%)	45 (20.27%)
4, n (%)	62 (27.93%)
5, n (%)	46 (20.72%)
6, n (%)	16 (7.21%)
7, n (%)	7 (3.15%)
8, n (%)	1 (0.45%)
Hemorrhage*	
Cerebral, n (%)	30 (13.51%)
Gastrointestinal, n (%)	20 (9.01%)
Other, n (%)	4 (1.80%)
HAS-BLED score	1.68 ± 0.86
0, n (%)	11 (4.95%)
1, n (%)	89 (40.09%)
2, n (%)	88 (39.64%)
3, n (%)	29 (13.06%)
4, n (%)	4 (1.80%)
5, n (%)	1 (0.45%)
Transthoracic echocardiography	
LA, mm	43.46 ± 5.63
RA, mm	44.09 ± 6.01
LVEDD, mm	48.00 ± 5.22
LVPW, mm	9.59 ± 1.97
IVS, mm	10.42 ± 2.16
LVEF, %	61.04 ± 7.99
MR, n (%)	
Moderate	25 (11.63%)
Severe	5 (2.33%)
TR, n (%)	
Moderate	35 (16.28%)
Severe	16 (7.44%)
LAA ostium diameter (mm)	
TEE	22.95 ± 4.15
DSA	22.89 ± 3.82

*One patient experienced both cerebral and gastrointestinal hemorrhage. BMI, body weight index; AF, atrial fibrillation; nPAF, non-paroxysmal atrial fibrillation; OAC, oral anticoagulation; HCM, hypertrophic cardiomyopathy; CAD, coronary artery disease; PAD, peripheral artery disease; PCI, percutaneous coronary intervention; CABG, coronary artery bypass graft; CKD, chronic kidney disease; LAA, left atrial appendage; LA, left atrium; RA, right atrium; LVEDD, left ventricular end diastolic diameter; LVPW, left ventricular posterior wall diameter; IVS, interventricular septum diameter; LVEF, left ventricular ejection fraction; MR, mitral regurgitation; TR, tricuspid regurgitation

### Procedure results

Table 2 listed the detailed procedure related data. WATCHMAN was the predominant LAAC device used in our center, which accounted for 217 (97.75%) of all the devices. Most of the procedures were performed under general anesthesia (168, 75.68%). Fifty-four (24.32%) procedures were performed awake with fluoroscopy guidance alone since 2020. Three cases failed because of the unsuitable LAA anatomy, making the device success rate 98.65%. The mean diameters of LAA measured by TEE or DSA were 22.94 mm and 22.90 mm, respectively. The mean compression rate of the LAAC device was 19.90% ± 5.52%, and no prominent PDL was overserved after fully deployed. Technical success was achieved in 219 cases, defined as the complete seal of LAA without device-related complications. Cardiac tamponade occurred in 2 cases. The mean activated clotting time was maintained at 362.91 s. However, three patients experienced an ischemic stroke within 3–12 h post-procedure. One occurred during the procedure, and the other happened 4 h after the end of implantation. Both patients had complete resolution of cardiac tamponade symptoms after pericardiocentesis and drainage. There were 6 cases of vascular complications, including 3 cases each of groin hematoma (> 6 cm) and femoral artery pseudoaneurysm formation (Table 3).

**Table 2:** Procedural data

Variables	All patients
LAAC brand	
WATCHMAN	217 (97.75%)
21 mm, n (%)	13 (6.00%)
24 mm, n (%)	39 (17.97%)
27 mm, n (%)	75 (34.56%)
30 mm, n (%)	51 (23.50%)
33 mm, n (%)	39 (17.97%)
Amplatzer Cardiac Plug	5 (2.25%)
22 mm	1 (20.00%)
24 mm	1 (20.00%)
27 mm	1 (20.00%)
28 mm	1 (20.00%)
30 mm	1 (20.00%)
Compression rate	19.90 ± 5.52%
General anesthesia, n (%)	168 (75.68%)
Minimalist approach, n (%)	54 (24.32%)
Concurrent catheter ablation of AF, n (%)	145 (65.32%)
Mean ACT values (s)	362.91 ± 88.51
Device size change, n (%)	1 (0.45%)
Recapture, n (%)	0 (0)
Immediate PDL, n (%)	
< 3 mm	220 (99.10%)
3–5 mm	2 (0.90%)
> 5 mm	0 (0)
Device success	219 (98.65%)
Technical success	219 (98.65%)
Procedural success	208 (93.69%)

LAAC, left atrial appendage closure; ACT, activated clotting time; PDL, peri-device leak

**Table 3:** Procedure related and 30 days postprocedural follow-up adverse events

Variables	All patients (n = 222)	Conventional approach (n = 168)	Minimalist approach (n = 54)	P value
Thromboembolism events, n (%)	3 (1.35%)	3 (1.78%)	0 (0)	> 0.999
Stroke, n (%)	3 (1.35%)	3 (1.78%)	0 (0)	> 0.999
TIA, n (%)	0 (0)	0 (0)	0 (0)	> 0.999
Other, n (%)	0 (0)	0 (0)	0 (0)	> 0.999
Pericardial effusion, n (%)	2 (0.90%)	1 (0.60%)	1 (1.85%)	0.428
Pericardial tamponade, n (%)	2 (0.90%)	1 (0.60%)	1 (1.85%)	0.428
Device embolization, n (%)	0 (0)	0 (0)	0 (0)	> 0.999
Device-related death, n (%)	0 (0)	0 (0)	0 (0)	> 0.999
Major bleedings, n (%)	0 (0)	0 (0)	0 (0)	> 0.999
Access-related complications, n (%)	6 (2.70%)	4 (2.38%)	2 (3.70%)	0.635
Groin hematoma > 6 cm, n (%)	3 (1.35%)	2 (1.19%)	1 (1.85%)	0.569
Femoral artery pseudoaneurysm, n (%)	3 (1.35%)	2 (1.19%)	1 (1.85%)	0.569
Arteriovenous fistula, n (%)	0 (0)	0 (0)	0 (0)	> 0.999

TIA, transient ischemic attack

### Anti-thrombotic regimen post procedure

**Fig. 3:**
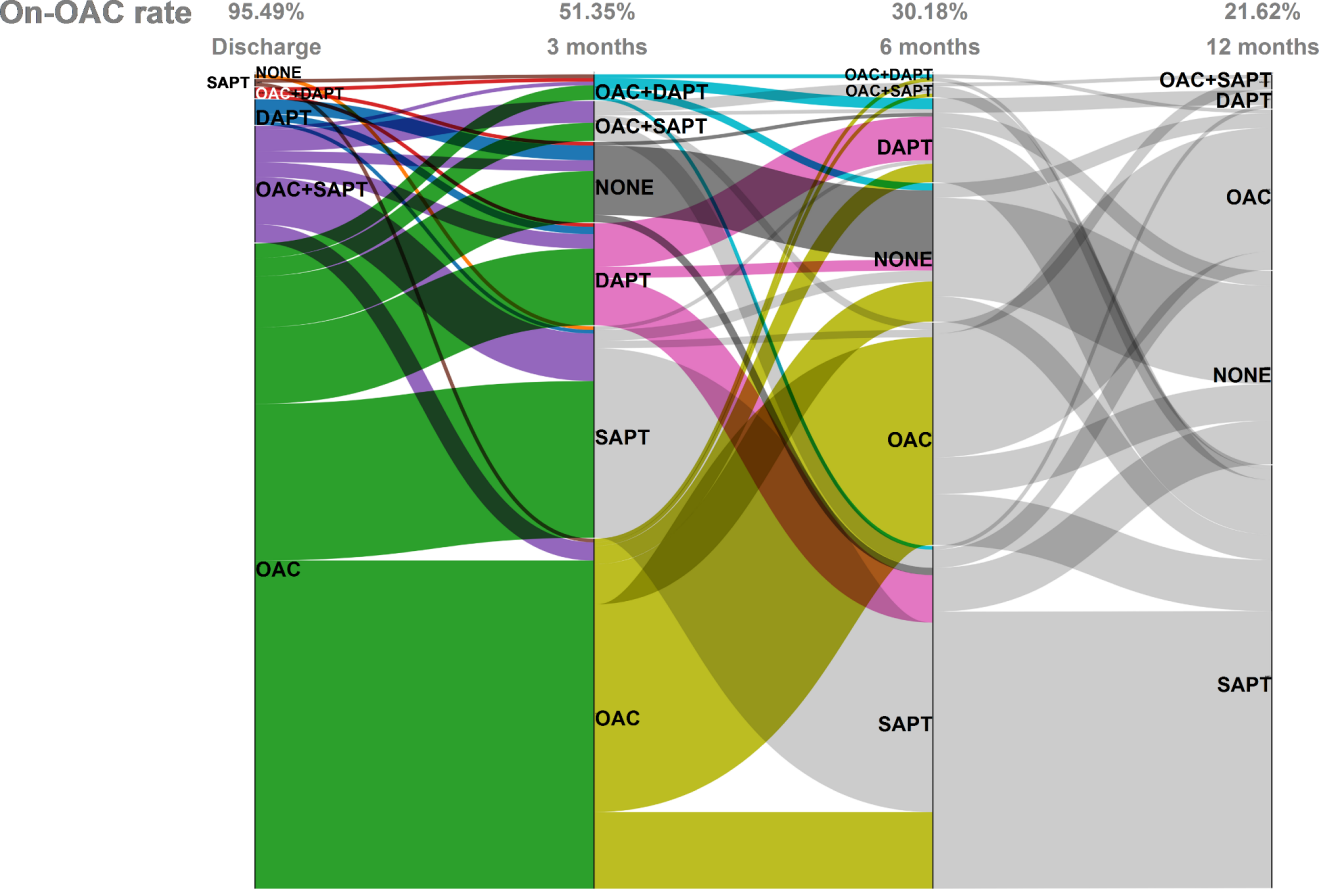
Change in anti-thrombotic regimen at discharge and 1 year post-implantation. SAPT, single antiplatelet therapy; DAPT, dual antiplatelet therapy; OAC, oral anticoagulation

The anti-thrombotic regimen post procedure was shown in Fig. 3. INR-adjusted warfarin or DOACs were prescribed after LAAC device implantation to prevent stroke or other thromboembolic events in nearly all patients (95.49%) at discharge. In only 1 patient with extreme high bleeding risk, none of the anticoagulant or antiplatelet drug were administered. At 3 months, half of the patients on-anticoagulation switched to either dual or single antiplatelet therapy. At 12 months post-procedure, oral anticoagulants were still being used in 21.62% of patients.

### Long-term outcomes

The data on long-term follow-up results is summarized in Table 4. A total of 609.43 person-years (PYs) follow up were achieved, with a median follow-up time of 2.30 years. The mean (SD) follow-up time was 2.81 (1.75) years. During these period, 26 patients presented with primary endpoint events, resulting in the primary endpoint incidence of 4.27 per 100 PYs. Cerebrovascular accident (CVA) and TIA were occurred in 19 patients (8.88%), with an incidence of 3.12 per 100 PYs. Seven patients die from cardiovascular disease with an incidence 1.15 per 100 PYs. Major bleeding events observed in 5 patients, with an incidence of 0.82 per 100 PYs. No hemorrhagic stroke was occurred. All patients completed the schedule reevaluation wthin the time frame. No PDL were found in the majority of cases (169, 78.97%). Only one patient (0.46%) exhibited a large PDL measuring 6 mm. For the remaining patients, the mean PDL size was 0.84 ± 1.08 mm. DRT was found in 5 patients (2.33%) by TEE or CCTA. In 1 patient, the DRT was found 1 year post implantation. Four of the DRT resolved after 30 to 90 days of anticoagulation, whereas one DRT resolved after six months of anticoagulation. None of the 5 patients developed stroke or thromboembolic event during the follow-up period. No significant discrepancies between the conventional approach and minimalist approach in terms of device-related thrombus (DRT) occurrences (2.98% vs. 0, p = 0.339) and substantial PDL (1.85% vs. 0, p = 0.243).

**Table 4:** Long-term follow up data

Variables	Patients with technical success (n = 214)
Primary efficacy endpoint	26 (12.15%)
Stroke/TIA	19 (8.88%)
Systemic embolism	2 (0.93%)
Death from cardiovascular causes	7 (3.27%)*
Severe bleeding events	5 (2.34%)
Cerebral hemorrhage, n (%)	0 (0)
Gastrointestinal bleeding, n (%)	3 (1.40%)
Other major bleeding, n (%)	2 (0.93%)
Minor bleeding events, n (%)	4 (1.87%)
Non-cardiovascular death, n (%)	15 (7.01%)
All-cause death, n (%)	19 (8.88%)
PDL > 5 mm, n (%)	1 (0.47%)
DRT, n (%)	5 (2.33%)

*Two patients also suffered stroke/TIA. TIA, transient ischemic attack; PDL, peridevice leak; DRT, device related thrombus

## Discussion

We reported here the clinical and procedural characteristics of patients with AF who underwent LAAC procedure in a real-world setting and the value of LAAC in preventing stroke and thromboembolic events, cardiovascular death, and bleeding among patients with AF. In general, this study showcases positive long-term outcomes of LAAC in NVAF patients. The LAAC procedure demonstrated effectiveness in reducing stroke risk, with low rates of adverse events and complications.

Aging is the key risk factor for AF and stroke [18]. In our study, the age distribution of patients was predominantly younger-old, consistent with previous studies from China [14, 19–21] yet younger than the reported results from two pivotal randomized studies and large registries [12, 13, 22, 23]. Despite patients in our study not being elderly, their CHA2DS2-VASc score was relatively higher and akin to other studies. Notably, we found that the percentage of patients with a previous history of stroke or systemic embolism was significantly higher than any of other studies, indicating a preference for those at high risk of thromboembolic events in the selection of eligible patients.

WATCHMAN was the first approved LAAC device in China, stood as the predominant choice for LAAC procedures in our center. Approximately a quarter of all interventions (24.32%) transitioning to a conscious sedation technique guided solely by fluoroscopy with the accumulation of experience. Comparisons between the conventional technique involving general anesthesia and the minimalist approach revealed comparable efficacy and safety profiles [15, 16]. Clinically irrelevant pericardial effusion was overserved in 2 patients at the end of the procedure, while 2 patients experienced cardiac tamponade, promptly relieved through pericardiocentesis. Compared to other studies, the slightly higher incidence of procedural complication in our group of patients might be attributed to the limited cases. The procedures performed by the minimalist approach resulted in one instance each of pericardial effusion and cardiac tamponade among the 54 cases, making it challenging to conclusively determine the relative safety of the minimalist approach compared to the conventional strategy. The RECORD study showed a low complication rate within 30 days of the procedure under local anesthesia and without TEE guidance, supporting the safety of the minimalist strategy. The shift towards resource-efficient strategies becomes increasingly significant, particularly in countries where healthcare systems burdened by limited resources. Moreover, adopting the minimalist approach offers the potential to curtail risks linked to ventilator-associated complications. The reduction in sedation levels has also been associated with a lower incidence of postoperative delirium. A cross-sectional study showed that interventional echocardiographers were exposed to higher radiation doses during LAAO procedures than interventional cardiologists [24]. Taking a simplified procedure strategy could also protect the interventional sonographer from radiation.

Two multicenter studies from China reported the data on the baseline and procedural characteristics. Zhai et al. pooled data from 658 Chinese patients who underwent WATCHMAN device implantation between 2014 and 2017, revealing a 97.7% success rate and a 0.6% procedure-related complication rate [25]. The RECORD study [14], involving 3096 patients from 39 hospitals, demonstrating a 97.9% implantation success rate in China, exceeding pivotal RCTs and featuring low peri-procedural complications and short-term adverse events (0.52% for the composite endpoint of death, stroke, and systemic embolism; 0.52% for life-threatening bleeding). Chiu et al. assessed the long-term outcomes of LAAC in Asian NVAF, revealing comparable ischemic stroke incidence between Watchman and ACP/Amulet groups (1.9 per 100 PYs vs. 1.4 per 100 PYs). The outcomes in terms of procedure success rate, efficacy, and adverse events were comparable to those reported in Caucasian populations. These findings suggest that LAAC can provide satisfactory outcomes and benefits for stroke prevention in Asia-Pacific NVAF patients with contraindications to oral anticoagulation therapy [26]. In line with the results from the RECORD trial and other recent published registry trials, the device success rate in our study was 98.65%. Only 3 device implantation was failed owing to the unsuitable LAA anatomy. With the accumulation of implantation experiences, in the remaining 219 patients, all the LAAC device was implanted successfully in the appropriate site with no > 5 mm jet between the LAA and device after deployment.

Observational studies and meta-analyses had suggested that it is feasible and safe to perform catheter ablation of AF concurrently with the LAAC procedure, the so-called “one-stop” strategy [27–29]. Given that our center is one of the largest centers for the treatment of cardiac arrhythmias in China, over 60% of the patients in our study underwent AF catheter ablation plus LAAC in a single procedure. Typically, AF catheter ablation preceded device implantation in the “one-stop” procedure. This sequence was designed to mitigate potential challenges to tissue-catheter contact caused by the occluder, particularly in areas like the left pulmonary and mitral isthmus. However, it’s worth noting that this sequence might induce myocardial edema, leading to an underestimated LAA ostium diameter, with subsequent development of extra peri-device leakage (PDL) as myocardial edema regresses within the first 3 months. In the TEE or CCTA re-evaluation, we did not observe a significant difference in terms of post procedural PDL between the “one-stop” group and the LAAC-alone group (p = 0.43). Also, we did not observe significant difference among the two groups during long-term follow-up period. All the same, it is still requiring more evidence to support this view.

Currently, no standardized anti-thrombotic regimen is available after LAAC implantation. The 2019 EHRA/EAPCI expert consensus statement on LAAC recommended that after WATCHMAN LAAC device implantation, patients with low bleeding risk should be given INR-adjusted warfarin or NOAC for 45 days, followed by clopidogrel (75 mg/d) for 6 months [17]. For patients who have contradiction to oral anticoagulation or at high risk of bleeding, DAPT was recommended for 1 to 6 months [17]. In our study, since the majority of patients were at high risk for thromboembolic event and only 15.53% were at high risk for bleeding, anticoagulants (DOACs or warfarin) were the most prescribed drugs post procedure. At 3 months, 6 months and 1 year, 51.35%, 30.18%, and 21.62% of the patients were still on anticoagulation therapy.

The presence of DRT after LAAC device implantation was associated with an increased risk of stroke and systemic embolism [30, 31]. The reported incidence of DRT post LAAC ranged from 1.6 to 16% [30, 32–34]. The highest incidence of DRT was reported in study [32]. In this study, up to 7.7% of patients were treated with neither anticoagulation nor antiplatelet therapy after LAA occlusion. Animal experiment had shown that by 45 days after implantation of the LAA occlusion device, a relatively complete endothelialization occurred in the device surface, suggesting that this could serve as a point of transition from anticoagulation to antiplatelet therapy [35]. Both the PROTECT-AF and PREVAIL study adjusted the anti-thrombotic regimen from aspirin plus warfarin to aspirin plus clopidogrel after excluding DRT by TEE at 45 days post procedure [12, 13]. However, we discovered 5 cases of DRT during follow-up with an incidence of only 2.33%. We speculated that the low incidence may be related to the high utilization rate of anticoagulants in this group of patients. Interestingly, all DRT were discovered beyond 45 days post LAAC, consistent with a meta-analysis that showed 85% of DRT presented after more than 45 days [31]. This may suggest that conventional imaging reevaluation strategy may underestimate the true incidence of DRT. Only 1 prominent PDL was identified in our study. This patient was treated with lifelong anticoagulation. However, a recent study found a slightly higher incidence of thromboembolism in patients with small PDL (> 0–5 mm) compared to those without [36]. This enlightened us that we should not simply assume the failure of the procedure by the extent of the PDL.

### Limitation

First, this is a single-center retrospective observational study with a small sample size and absence of a control group. Nonetheless, this is the first report of longest-term follow-up data in Chinese patients who underwent LAAC. Second, the number of cases of LAA occlusion using a disc occlusion device is small and we cannot compare it with the WATCHMAN LAAC device. Third, owing to the bias of patient selection, the anticoagulation regimen adopted in this study may not be suitable for generalization.

## Conclusion

This study was the first to report long-term follow-up results after LAAC in a Chinese population. The study demonstrates that LAAC is a safe and effective alternative option for Chinese patients with AF, with a high success rate, few complications as well as less long-term adverse outcome events.

### Electronic supplementary material

Below is the link to the electronic supplementary material.

**Supplementary Material 1:** WATCHMAN Access Sheath Advancement into LSPV

**Supplementary Material 2:** LAA Cineangiography

**Supplementary Material 3:** Insertion of the Delivery System

**Supplementary Material 4:** Device Deployment

**Supplementary Material 5:** PASS Criteria Assessment

**Supplementary Material 6:** Device Release

## Data Availability

The data that support the findings of this study are available from the corresponding author upon reasonable request.
