# Supplementary material for: Characteristics and outcome of patients with left atrial appendage closure in China: a single-center experience
Source: BMC Cardiovasc Disord. 2024 Feb 14;24:108. doi: 10.1186/s12872-023-03651-8 (PMC10865656; doi:10.1186/s12872-023-03651-8)
Supplement: Supplementary file 6 — Supplementary Material 6: Device Release [file 12872_2023_3651_MOESM6_ESM.docx]

**Supplementary**

**I. Pre-Deployment**

Following the completion of atrial fibrillation radiofrequency ablation, a super-stiff guidewire is guided under fluoroscopy into the left superior pulmonary vein (LSPV). The guidewire is secured in position, and the transseptal sheath is slowly withdrawn from the left atrium. The WATCHMAN access sheath is flushed with heparinized saline. The sheath and dilator were assembled coaxially. Under fluoroscopic guidance, the WATCHMAN access sheath is advanced slowly over the super-stiff guidewire to the orifice of the LSPV, after which the dilator and super-stiff guidewire were withdrawn (Figure 1).

**Figure 1. WATCHMAN Access Sheath Advancement into LSPV**


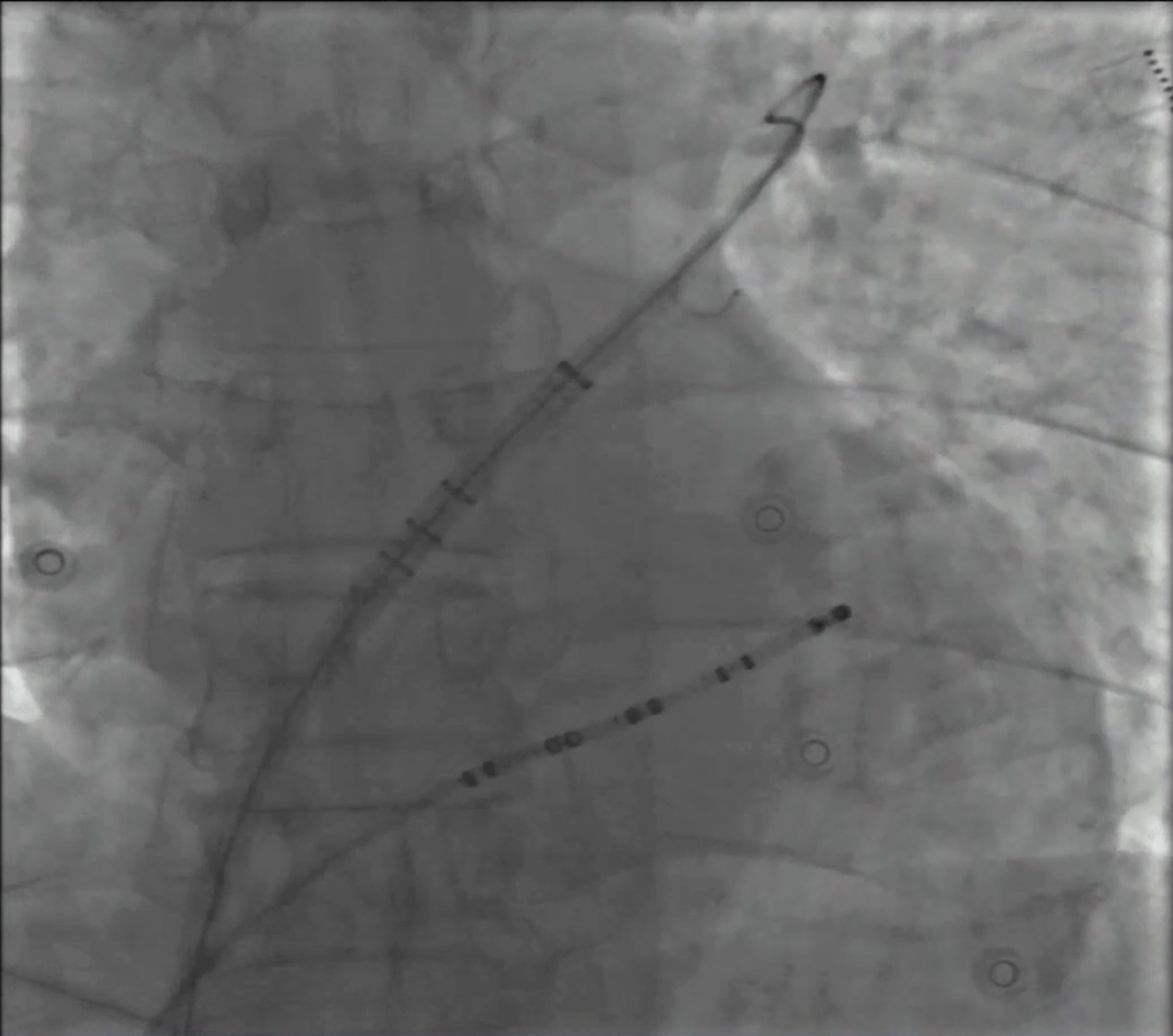


Next, a 6 Fr pigtail catheter, previously aspirated and flushed with heparinized saline, was introduced through the WATCHMAN access sheath into the LSPV. The sheath and pigtail catheter are slowly withdrawn, and the pigtail catheter is rotated and advanced into the deepest lobe of the LAA for LAA cineangiography, using the coronary mode at a frame rate of 15 frames/second. Typically, LAA cineangiography is performed in the RAO projection between 30° and 45° and CAU projection at approximately 20° to ensure thorough opacification of the various lobes within the LAA, providing a clear delineation of the LAA’s morphology. The angles may vary among patients to obtain clear images of the LAA, and adjustments may be made as necessary. After LAA cineangiography, measurements of the LAA orifice diameter and depth are taken to determine the appropriate device size (Movie 1).

**II. Deployment**

1. Selection of the WATCHMAN Device Size: The selection of the WATCHMAN Device (Boston Scientific, USA) is generally based on the maximum orifice diameter plus an additional 4-6 mm. In special cases where the LAA orifice is elliptical, highly irregular, or exhibits significant contractions, pre-procedure TEE or CTA may be considered to guide device selection (Figure 2).

**Figure 2. Selection of the WATCHMAN Device Size**


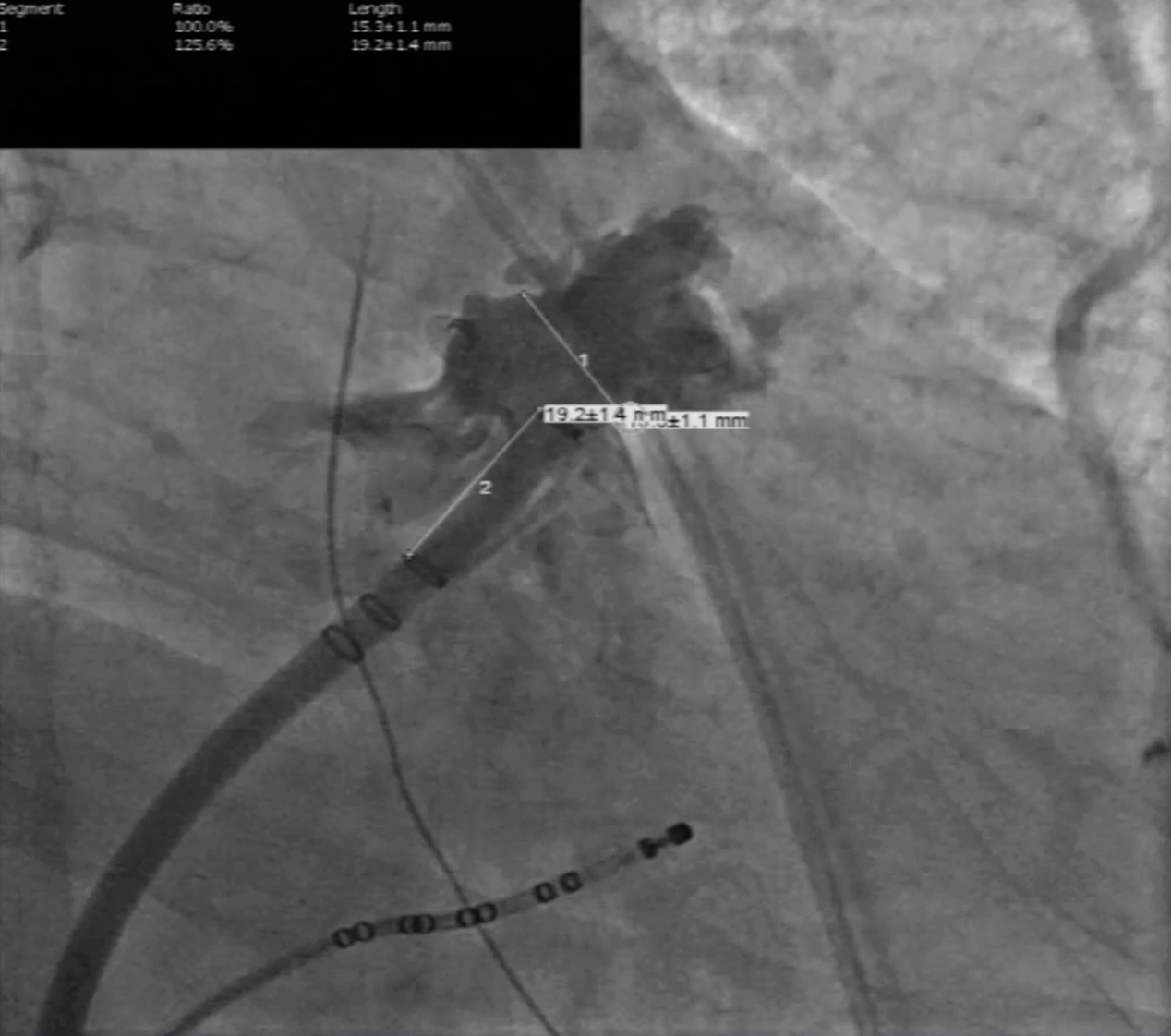


2. Withdrawal of the Pigtail Catheter and Insertion of the Delivery System: Under fluoroscopy guidance, the access sheath is advanced along the pigtail catheter as far distally as safely possible. The pigtail catheter is then gently removed to ensure that the axis and position of the access sheath remain unchanged. After confirming smooth blood backflow through the access sheath, the continuously flushed delivery system is inserted into the WATCHMAN access sheath. The marker band of the delivery system is aligned with the marker ring at the far end of the access sheath. The delivery system is secured in place, the access sheath is withdrawn, and the two components are locked together (Movie 2).


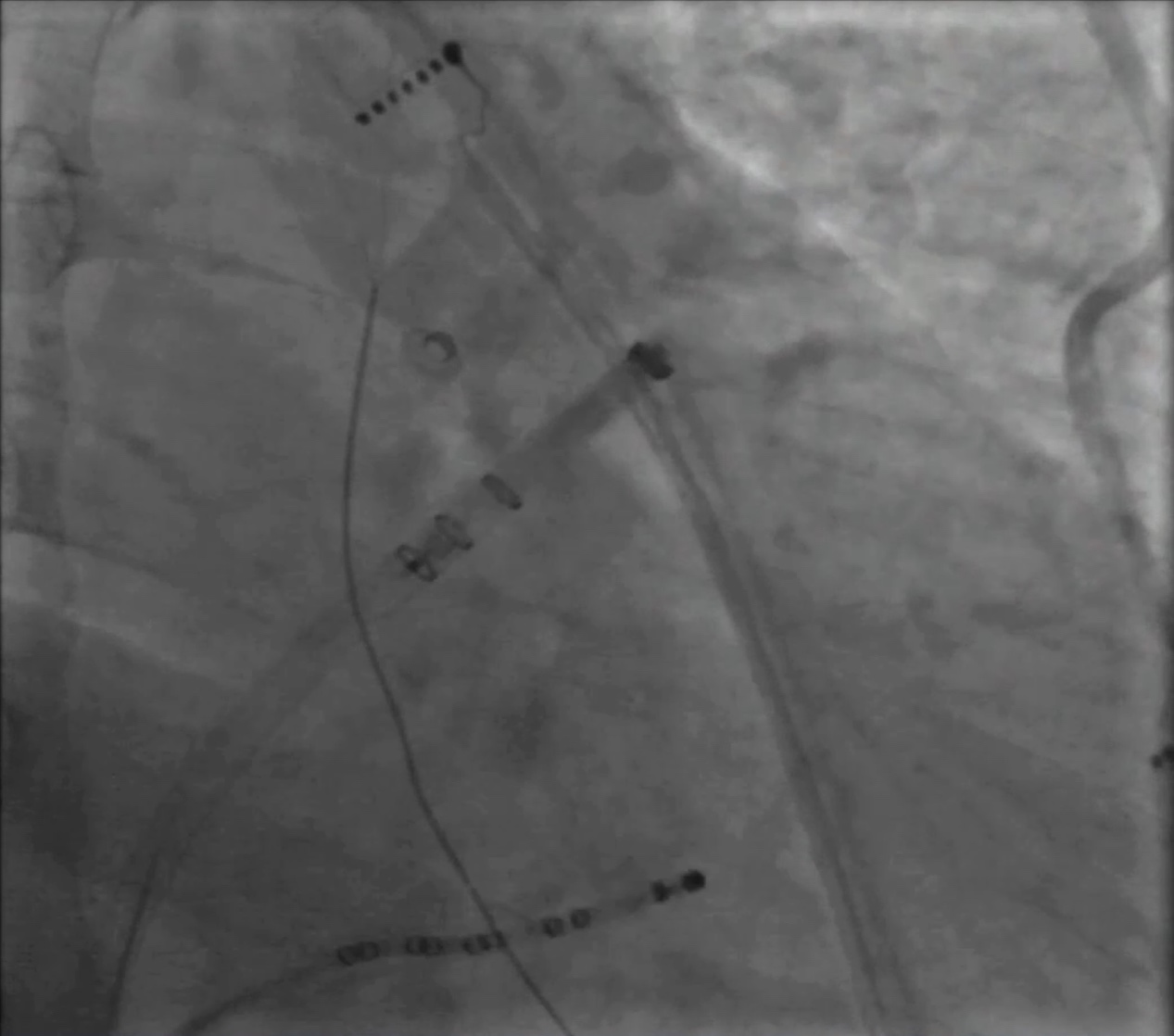


3. Device Deployment: Device deployment is typically performed in the RAO 30° and CAU 20° positions, guided exclusively by fluoroscopy. In specific cases, alternative angles that provide the clearest view of the distal LAA and depth are also employed. To deploy the device, every effort is made to maintain the device's shoulder in a relatively fixed position relative to the working line. As the access sheath is gradually retracted, the device's feet are unfolded. The delivery catheter may also be gently withdrawn to align with the intended deployment position. As the device begins to unfold, a slight forward tension is maintained to prevent migration towards the LAA ostium (Movie 3).

4. PASS Criteria Assessment: Following device deployment, the PASS criteria are assessed under fluoroscopy alone (Movie 4):

P (Position): Confirmation that the device's shoulders are correctly positioned at the LAA orifice, typically viewed in RAO 30° and CAU 20° to assess the relationship between the device's upper and lower edges and the LAA orifice's position.

A (Anchor): Verification of device stability after gently rotating the release handle counterclockwise. An appropriate degree of pulling is applied to the release handle and released. This process is observed for synchronous movement of the device with the atrial wall and significant device rebound without positional changes, indicating device stability. Additional traction can be performed in cases where device stability is in question.

S (Size): Measurement of the compression ratio under fluoroscopy alone. Radiographs are taken in the RAO 30° and CRA 20° positions to determine the device's maximum diameter for calculating the compression ratio.

S (Seal): Assess for the presence of peri-device leaks (PDL) following device deployment. Under fluoroscopy, three positions imaging is conducted, inspecting the relationship between the device's edges and the LAA orifice. Ensure that no contrast agent enters the LAA around the device. If a PDL of ≥5 mm is observed, the device's position should be adjusted or replaced. Assessment for the presence of peri-device leaks (PDL) after device deployment. fluoroscopy is performed in three different views (RAO 30° + CRA 20°, RAO 30° + CAU 20°, and tangential RAO 30°) to examine the relationship between the device's edges and the LAA orifice. Ensure that no contrast agent enters the LAA around the device. If a PDL of ≥5 mm is observed, device position adjustments or replacements should be carried out.

6. Release: Upon confirming compliance with the PASS criteria, the delivery sheath is advanced and held against the device. The release handle is rotated counterclockwise by 3-5 turns to complete device release (Movie 5).

7. Post-Release Assessment: Another cineangiography is performed to evaluate the position and morphology of the device after release.
